# A missed opportunity: receipt of medication for opioid use disorder in Southern California acute care settings

**DOI:** 10.1186/s13722-026-00703-0

**Published:** 2026-07-16

**Authors:** Brian Maila, Carla Marienfeld

**Affiliations:** 1 Division of Global Public Health, University of California San Diego, 9500 Gilman Drive, La Jolla, San Diego, CA 92093 USA; 2 School of Social Work, San Diego State University, 5500 Campanile Drive, San Diego, CA 92182 USA; 3 Department of Psychiatry, University of California San Diego, 9500 Gilman Drive, La Jolla, San Diego, CA 92093 USA

**Keywords:** Medications for opioid use disorder, Opioid-related diagnosis, Opioid use disorder, Acute care settings, In-patient, Emergency department, Substance-related diagnosis

## Abstract

**Background:**

Medications for opioid use disorder (MOUD) reduce overdose risk and improve outcomes, yet receipt remains low in acute care settings, including among patients engaged in health care. Understanding sociodemographic and clinical factors associated with lower odds of MOUD receipt is essential to inform strategies that expand access and strengthen responses to the opioid epidemic in the United States.

**Objective:**

To describe sociodemographic and clinical characteristics and estimate factors associated with lower odds of MOUD receipt among patients with opioid-related diagnoses (ORD) care involvement who had an assigned primary care provider and who utilized acute care settings within a large academic health system in Southern California.

**Methods:**

We analyzed electronic health records from 4,242 patients who had ≥ 1 acute care encounter and a UC San Diego Health-assigned primary care provider between April 22, 2022, and April 21, 2025. Patients were classified as having ORD clinical involvement if they had a documented ICD-10 ORD, an MOUD prescription within 6 months prior to the index acute care encounter, an MOUD prescription during the index acute care encounter, or an opioid overdose-related encounter. Descriptive statistics characterized the sample; logistic regression estimated odds of limited MOUD receipt.

**Results:**

Among patients with opioid-related involvement who had an assigned primary care provider and utilized acute care services, 428 (10.1%) received MOUD during the acute care encounter. Prior MOUD prescription within six months preceding the index acute care encounter was associated with the highest odds of MOUD receipt (aOR = 24.11, 95% CI [18.60, 31.53], *p* < 0.001). Having other substance-related diagnoses was also associated with higher odds of MOUD receipt (aOR = 1.85, 95% CI [1.29, 2.69], *p* < 0.001). In contrast, patients with any mental health diagnosis had lower odds of MOUD receipt (aOR = 0.73, 95% CI [0.57, 0.94], *p* = 0.02). Although overdose-related encounters were associated with lower odds of MOUD receipt (aOR = 0.56, 95% CI [0.29, 1.00], *p* = 0.06), the association did not reach statistical significance after adjustment.

**Conclusion:**

Significant gaps persist in MOUD receipt among patients with opioid-related involvement in acute care settings. Prior MOUD prescription was strongly associated with MOUD receipt, suggesting barriers in receipt among patients without previous prescriptions. Patients with mental health comorbidity had lower odds of MOUD receipt. Although overdose-related encounters were associated with lower adjusted odds of MOUD receipt, additional research is needed to better understand barriers to MOUD initiation following overdose-related encounters. Overall, these findings call for tailored strategies such as addiction consultation services, peer navigation programs, integrated addiction and behavioral health services, and low-barrier MOUD initiation protocols within acute care settings.

**Supplementary Information:**

The online version contains supplementary material available at https://doi.org/10.1186/s13722-026-00703-0.

## Introduction

Opioid use—alone or combined with other substances—remains a leading driver of preventable overdose-related morbidity, mortality, and emergency department utilization in the United States [1–5]. Notwithstanding the negative consequences of the opioid crisis, effective, evidence-based treatment is available and represents the standard of care for most patients with opioid-related diagnoses (ORDs) in acute care settings [1]. ORDs encompass a range of conditions related to opioid use, including opioid use disorder (OUD), opioid intoxication, withdrawal, and opioid-induced mental health disorders. Medications for opioid use disorder (MOUD)—including buprenorphine, methadone, and naltrexone—are the gold standard, consistently demonstrating reductions in overdose deaths, improved treatment retention, decreased behaviors associated with infectious disease transmission (e.g., HIV and hepatitis C virus), and lowering healthcare costs [6–12]. However, much of this evidence reflects MOUD use across outpatient and community-based settings rather than initiation during acute care encounters. Despite these benefits, MOUD utilization remains limited, with only 1 in 5 individuals with an ORD receiving these medications across sociodemographic groups and clinical settings [1]. Moreover, relatively fewer studies have examined factors associated with MOUD initiation or receipt specifically within acute care settings. Therefore, identifying factors associated with lower receipt of MOUD during acute care encounters is essential for informing tailored interventions and ensuring appropriate care for patients with ORDs, thereby supporting ongoing national efforts to address the opioid epidemic.

Acute care settings offer critical opportunities to initiate and expand access to MOUD, yet they remain underutilized [13]. Encounters in these settings often occur during crises—such as nonfatal overdose, withdrawal, or opioid-related complications—when patients may be especially receptive to treatment initiation [14, 15]. Although research demonstrates the feasibility of initiating MOUD in the emergency department (ED), implementation and uptake are inconsistent [13, 16, 17]. In response, California has implemented several statewide initiatives to expand access to MOUD, including the California Bridge (CA Bridge) program, which supports emergency department–initiated treatment and linkage to ongoing care, as well as Department of Health Care Services (DHCS) opioid response efforts aimed at increasing treatment capacity across healthcare settings [18, 19]. In inpatient hospitalizations, providers cite structural and logistical challenges—including limited MOUD prescribers, uncertainty around outpatient linkage, and competing clinical demands—as barriers to initiation [20, 21]. Even with statewide initiatives such as California’s CA Bridge program, substantial variation persists across hospitals, indicating that system-level, sociodemographic, and clinical factors significantly influence MOUD receipt [22]. Thus, identifying the sociodemographic and clinical factors that are associated with lower odds of MOUD receipt in acute care settings is essential for informing interventions that expand access.

Despite strong evidence supporting the feasibility and effectiveness of acute care–initiated MOUD, receipt remains low and uneven across patient groups. Racial and ethnic disparities are well documented: Black or African American and Native Hawaiian/Pacific Islander patients are less likely than White patients to receive buprenorphine [13, 23], though some studies report lower odds among White patients [24] or no racial differences [25]. Gender-based differences also appear, with women less likely to receive MOUD in some studies [23, 24], while others report no gender differences [25]. Insurance status further shapes access, with self-pay patients having lower odds of MOUD receipt compared with Medicaid beneficiaries [25]. Beyond sociodemographic factors, clinical presentation and prior treatment history also influence prescribing patterns, particularly in the context of evolving drug supplies and policy environments [23, 25]. Collectively, this evidence shows that patient-level characteristics play a critical yet insufficiently understood role in determining which patients fail to receive MOUD in acute care settings.

Our study examines patient-level factors associated with lower odds of MOUD receipt among patients with ORD care involvement in Southern California acute care settings using electronic health records (EHRs). Given the importance of capturing longitudinal care and prescribing data within the EHR, this study focuses on patients with opioid-related diagnoses who had an assigned primary care provider and utilized acute care services within the health system. By examining sociodemographic and clinical determinants, we identify characteristics independently associated with lower MOUD receipt, providing insight into factors that contribute to inconsistent treatment uptake within these settings. Specifically, our aims were to: (I) describe the sociodemographic and clinical characteristics of patients with opioid-related diagnoses care involvement who had an assigned primary care provider and utilized acute care services; and (II) estimate the odds of sociodemographic and clinical factors associated with lower odds of MOUD receipt. Findings from this work provide an evidence base for tailoring acute care protocols, improving MOUD receipt, and strengthening the role of acute care encounters as a critical entry point for initiating life-saving treatment and evidence-based treatment for ORD.

## Materials and methods

### Sample and data source

This cross-sectional study used de-identified EHR data from UCSD Health’s Substance-Related Diagnosis (SRD) Registry in Southern California. The index acute care encounter was defined as the first eligible acute care encounter (emergency department encounter or inpatient hospitalization) for each patient during the study period (April 22, 2022–April 21, 2025). The study included patients with opioid-related involvement who had at least one acute care encounter and a UCSD-assigned primary care provider (PCP) during the study period.

Opioid-related involvement was defined as: (a) an opioid-related diagnosis (ORD), operationalized using ICD-10-CM F11.xx codes; (b) receipt of MOUD within six months prior to or during the index acute care encounter; or (c) an opioid overdose-related encounter (Supplementary File 2). This combined approach was used to improve ascertainment of clinically relevant opioid-related involvement because reliance on diagnostic codes alone may underestimate the true prevalence of opioid involvement due to under-coding and inconsistent coding practices, particularly when clinical attention is focused on comorbid conditions [26–29]. Algorithms that incorporate diagnosis, treatment, and overdose information have demonstrated improved performance in identifying opioid involvement and are recommended by the Centers for Disease Control and Prevention (CDC) and the Council of State and Territorial Epidemiologists (CSTE) for opioid surveillance [30, 31]. Additionally, the sensitivity and specificity of ORD ICD codes are imperfect [32], and these codes remain underutilized or inconsistently applied in clinical practice [33].

ORDs were defined using ICD-10-CM F11.xx codes and included opioid use disorder (OUD), opioid intoxication, opioid withdrawal, and opioid-induced mental and behavioral disorders. A UCSD-assigned primary care provider (PCP) was required to ensure availability of outpatient prescribing and encounter data within the EHR, including MOUD receipt in the six months prior to the index acute care encounter. Because the health system is not a closed network and does not capture care received outside UCSD Health, this restriction was necessary to reliably assess prior treatment exposure and defines a study population of patients with opioid-related involvement who had an assigned PCP and utilized acute care services. SRDs in the registry include substance use disorders, intoxication, withdrawal, overdose, and general substance use. Supplementary File 1 provides the SRD categories and corresponding ICD codes. Data were extracted from the EHR by the Data Extraction Concierge Service team at the Altman Clinical and Translational Research Institute and securely delivered through a Health Insurance Portability and Accountability Act (HIPAA)-compliant Virtual Research Desktop following approval of the UCSD IRB protocol and associated data request.

### Ethics and IRB approval

This study was approved by the UCSD IRB [Protocol #811762]. There was no interaction with human subjects. The study used de-identified data, qualifying the project as minimal risk and exempt under federal guidelines for research on existing data. Data management followed institutional and HIPAA standards.

### Measures

#### Dependent variable

The outcome measured in this study was receiving any MOUD prescription during the index acute care encounter within the data extraction period from the EHR. MOUD receipt during the index acute care encounter was defined as the presence of a documented prescription order for MOUD (buprenorphine, methadone, naltrexone, or extended-release formulations) during the encounter, including both emergency department encounters and inpatient hospitalizations. Methadone prescribed for pain management and dispensed through community pharmacies was not included as MOUD in this study. Only methadone documented as part of opioid-related diagnosis care involvement within the acute care setting was included in the MOUD definition. This definition includes both newly initiated MOUD and prescription orders reflecting continuation of previously prescribed MOUD (e.g., from outpatient or primary care settings). Patients with a prior MOUD prescription were classified as having MOUD receipt (Yes) only if a MOUD prescription order was documented during the index acute care encounter; prior prescriptions alone were not sufficient for classification. Extended-release formulations (e.g., long-acting injectable buprenorphine and naltrexone) were included and classified as MOUD receipt when a prescription order was documented during the encounter. This variable was classified as Yes if MOUD was received and No if not. The EHR data do not consistently distinguish initiation from continuation; therefore, MOUD receipt is modeled as a unified outcome.

#### Independent variables

Independent variables were chosen based on prior literature [3] and clinical relevance, covering both sociodemographic and clinical characteristics. Age was measured in years and grouped into four categories: 18–34, 35–49, 50–64, and ≥ 65 years. Sex was coded as Male and Female. To reduce sparse cells and improve model parsimony, race and ethnicity were combined into a composite race/ethnicity variable with four categories: Non-Hispanic White, Non-Hispanic Black, Hispanic/Latinx, and Other/Mixed/Unknown. The Other/Mixed/Unknown category included individuals identifying as Asian, American Indian/Alaska Native, Native Hawaiian/Pacific Islander, multiracial, other race, or unknown race/ethnicity. Marital status was categorized as Single, Married/Partnered, and Previously Married/Other. Insurance type was categorized as Commercial (commercial/motor vehicle insurance/workers compensation/other managed care), Medicare (Medicare/Medicare managed care), Medicaid/Public (Medicaid-California/Medicaid-out-of-state/Medicaid managed care/County medical services and Other Government plans such as Tricare/Federal Plans), and Self-Pay.

Key clinical variables—any mental health diagnosis (ICD codes in Supplementary File 1), other substance-related specific diagnosis, prior outpatient visit, MOUD prescription 6 months prior to the acute care encounter, and overdose-related encounter—were binary (No/Yes). Prior outpatient visit was included as a proxy measure of recent healthcare engagement. Given that MOUD may be prescribed in outpatient settings without a corresponding documented primary care provider (PCP) encounter, this variable was defined to include both PCP visits and MOUD prescriptions within the six months preceding the index acute care encounter. This composite definition improves capture of outpatient care engagement and prior treatment exposure, which are clinically relevant factors that may influence MOUD receipt during acute care encounters. However, the outpatient visit variable was ultimately excluded from adjusted analyses because of conceptual overlap with prior MOUD prescription and concerns regarding multicollinearity.

“Other substance-related specific SRDs” was additionally defined as a composite variable representing the presence of non-opioid substance-related diagnoses, including alcohol, cannabis, sedative/hypnotic/anxiolytic, cocaine, other stimulant, hallucinogen, nicotine, inhalant, and other psychoactive substance-related disorders, identified using ICD-10-CM codes (F10.xx–F19.xx; excluding F11.xx) as detailed in Supplementary File 1. Opioid overdose–related encounters were identified using ICD-10-CM poisoning codes (T40.x as indicated in Supplementary File 1), including sequela codes, and represent non-fatal overdose–related clinical encounters documented during emergency department encounters or inpatient hospitalizations. These codes capture both acute overdose events and subsequent encounters related to prior overdose. Fatal overdoses were not captured in this dataset. The Charlson Comorbidity Index (CCI) was modeled as a continuous measure of comorbidity burden to improve model stability and preserve information across levels of medical complexity. Sensitivity analyses additionally evaluated models excluding overdose-related encounter from adjusted analyses to assess the potential influence of sparse cells associated with overdose-related encounters.

### Data analysis

Data management was conducted using IBM SPSS Statistics for Windows, Version 30.0 (IBM Corp., Armonk, NY, USA) [34], and all statistical analyses were performed in R version 4.6.0 (R Foundation for Statistical Computing, Vienna, Austria, 2026) [35], with statistical significance defined as a two-sided α = 0.05. A complete-case analysis approach was used, retaining only patients with complete data on all variables included in the analyses. Descriptive analyses summarized sociodemographic and clinical characteristics, with categorical variables reported as frequencies and percentages and continuous variables summarized using means and standard deviations. Bivariate analyses compared patients who received MOUD with those who did not during the index acute care encounter, using chi-square (χ²) tests for categorical variables and independent samples t-tests for continuous variables.

Bivariate logistic regression was then conducted, entering each predictor individually with MOUD prescription (Yes vs. No) as the binary dependent variable, reporting odds ratios (ORs), 95% confidence intervals (CIs), and p-values. A multivariable logistic regression model followed to assess adjusted associations between participant characteristics and MOUD prescription, including all variables with theoretical relevance and clinical importance. Adjusted odds ratios (aORs), 95% CIs, and p-values are reported. To improve model parsimony and reduce concerns regarding sparse cells and overparameterization, several variables were collapsed into broader clinically meaningful categories prior to multivariable modeling. Age was categorized into four groups (18–34, 35–49, 50–64, and 65 + years), race and ethnicity were combined into a composite race/ethnicity variable, and marital status and insurance categories were simplified. The Charlson Comorbidity Index was modeled continuously. The outpatient visit variable and the LACE Index were excluded from adjusted analyses because of conceptual overlap and multicollinearity concerns. Model diagnostics included assessment of multicollinearity using variance inflation factors (VIFs), calibration using the Hosmer–Lemeshow goodness-of-fit test, and discrimination using the area under the receiver operating characteristic (ROC) curve.

The primary analytic model used a broad opioid-related involvement definition that included patients with opioid-related diagnoses (ORDs), prior MOUD prescription, MOUD receipt during the acute care encounter, or overdose-related encounters (*N* = 4,242). Sensitivity analyses evaluated alternative sample definitions and model specifications, including a narrower opioid-related involvement definition consisting of patients with ORDs and/or MOUD receipt during the acute care encounter or overdose-related encounters (*N* = 3,919), and a more restrictive EHR-based definition limited to ORDs and/or overdose-related encounters documented in the EHR (*N* = 3,585). Additional sensitivity analyses also evaluated models excluding overdose-related encounter from adjusted analyses to assess the potential influence of sparse cells associated with overdose-related encounters. For each model specification, bivariate and multivariable logistic regression analyses were conducted, and adjusted estimates were compared across models to evaluate consistency in the direction, magnitude, and statistical significance of associations.

## Results

### Description of the sample included in the analysis

**Fig. 1:**
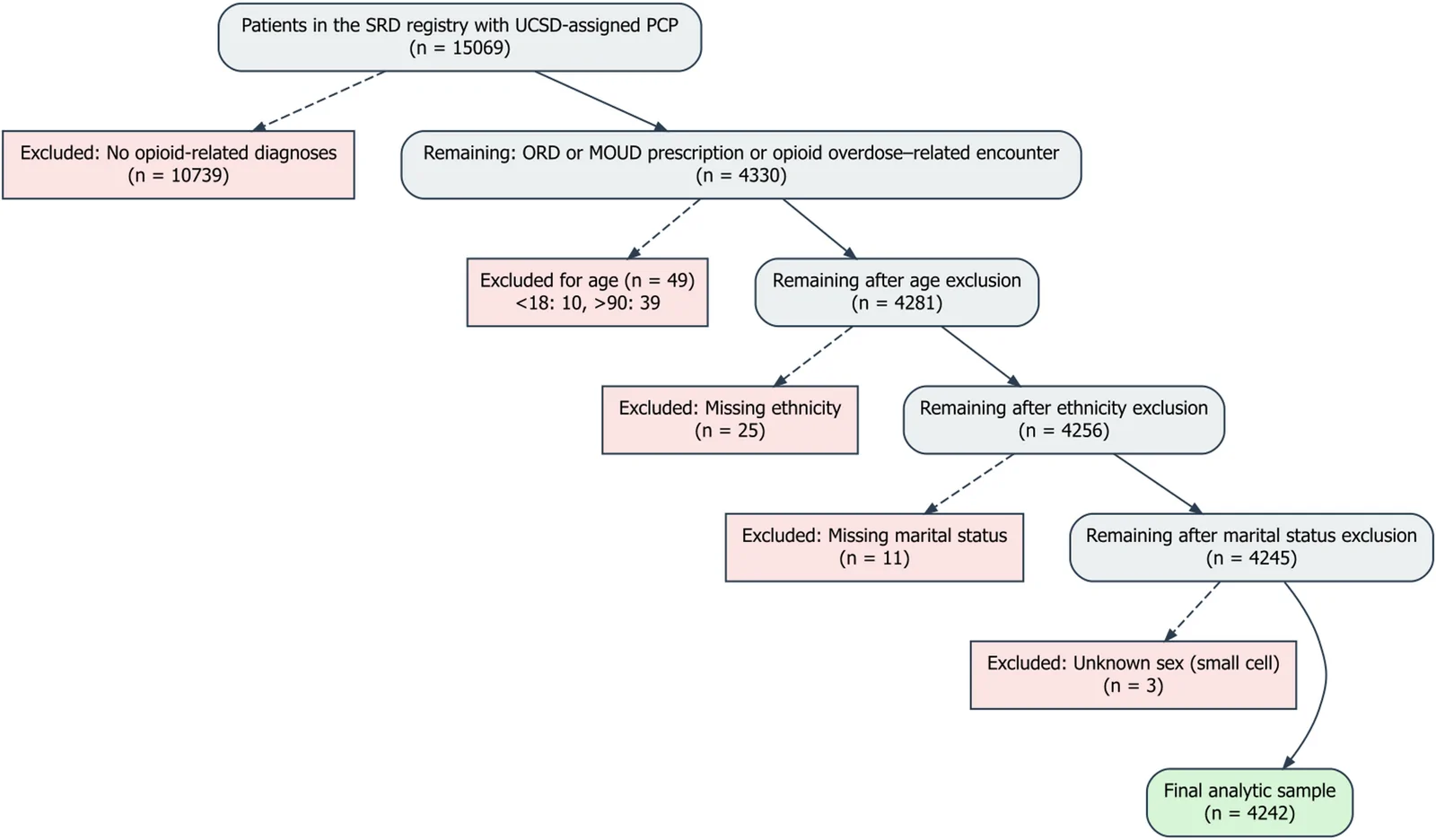
Flow diagram of sample selection. Final analytic sample=Those with ORD; Those who Received an MOUD prescription; Those who had an opioid overdose-related encounter, all aged 18–90 and with a UCSD-assigned PCP

A total of 15,069 patients had an acute care encounter, an assigned PCP at UCSD, and a documented ICD-10 substance-related diagnosis (SRD) from April 22, 2022, to April 21, 2025, as recorded in the SRD Registry within the electronic health records (EHR) system of UC San Diego Health, a large academic medical center in Southern California. Patients without an opioid-related diagnosis (ORD) were excluded, resulting in a final sample of 4,330 patients with evidence of opioid-related involvement. Out of the 4,330 patients, we excluded 49 because they were below the age of 18 and above the age of 90. First, exclusions due to missing data (*N* = 36; 0.84%) for any variables required in the analysis were performed, including 25 patients (0.58%) with missing data on ethnicity, and 11 patients (0.26%) with missing data on marital status, leaving the number of patients at 4,245. Then exclusions were conducted for categories with small numbers on categorical variables, particularly 3 patients (0.07%) who identified as unknown on the sex variable, to avoid skewing the estimates during the analysis, leaving the total number of records at 4,242. Therefore, a total of 4,242 patients remained in the final analytic sample, all of whom had had an ORD or an MOUD prescription 6 months prior to the index acute care encounter or MOUD prescription during the index acute care encounter or opioid overdose-related encounter, were between ages 18 and 90, had an assigned UCSD PCP, and had complete data on all variables required for analysis (Fig. 1).

### Sample sociodemographic and clinical characteristics by medication for opioid use disorder status

Table 1 summarizes the sociodemographic and clinical characteristics of the study sample by receipt of medications for opioid use disorder (MOUD) during the index acute care encounter. Of the 4,242 patients included, 428 (10.1%) received MOUD and 3,814 (89.9%) did not. Patients receiving MOUD were younger on average than those not receiving MOUD (mean age 46.5 vs. 50.3 years, *p* < 0.001), with larger proportions of MOUD receipt observed among individuals aged 18–49 years. Differences were also observed by marital status and insurance type, with the MOUD group including larger proportions of individuals who were single and self-pay, while the proportion covered by Medicare was smaller. Several clinical characteristics also differed between groups. The MOUD group had larger proportions of patients with other substance-related diagnoses, prior outpatient visits within the previous six months, and prior MOUD prescriptions (all *p* < 0.001). In contrast, the proportion of patients with overdose-related encounters was smaller among patients receiving MOUD (3.0% vs. 8.7%). Patients receiving MOUD also had lower Charlson Comorbidity Index scores on average, indicating lower comorbidity burden. No statistically significant differences were observed by sex or race/ethnicity.

**Table 1:** Sociodemographic and clinical characteristics of sample stratified by medication for opioid use disorder status

Variable	Level	Overall	No MOUD	Any MOUD	*P*-value
All, n (%)		4,242 (100%)	3,814 (89.9%)	428 (10.1%)	-
Age, mean (SD)		49.9 (16.7)	50.3 (16.7)	46.5 (16.6)	< 0.001
Age Group, n (%)					0.001
	18–34	984 (23.2%)	865 (22.7%)	119 (27.8%)	
	35–49	1,132 (26.7%)	1,003 (26.3%)	129 (30.1%)	
	50–64	1,123 (26.5%)	1,016 (26.6%)	107 (25.0%)	
	65+	1,003 (23.6%)	930 (24.4%)	73 (17.1%)	
Sex, n (%)					0.26
	Male	2,469 (58.2%)	2,209 (57.9%)	260 (60.7%)	
	Female	1,773 (41.8%)	1,605 (42.1%)	168 (39.3%)	
Marital Status, n (%)					< 0.001
	Single	2,411 (56.8%)	2,132 (55.9%)	279 (65.2%)	
	Married/Partnered	1,027 (24.2%)	934 (24.5%)	93 (21.7%)	
	Previously Married/Other	804 (19.0%)	748 (19.6%)	56 (13.1%)	
Race/Ethnicity, n (%)					0.54
	Non-Hispanic White	2,245 (52.9%)	2,025 (53.1%)	220 (51.4%)	
	Non-Hispanic Black	468 (11.0%)	412 (10.8%)	56 (13.1%)	
	Hispanic/Latinx	984 (23.2%)	888 (23.3%)	96 (22.4%)	
	Other/Mixed/Unknown	545 (12.8%)	489 (12.8%)	56 (13.1%)	
Insurance Type, n (%)					0.004
	Commercial	1,757 (41.4%)	1,576 (41.3%)	181 (42.3%)	
	Medicare	586 (13.8%)	548 (14.4%)	38 (8.9%)	
	Medicaid/Public	574 (13.5%)	521 (13.7%)	53 (12.4%)	
	Self-Pay	1,325 (31.2%)	1,169 (30.7%)	156 (36.4%)	
Any Mental Health Diagnosis, n (%)		2,644 (62.3%)	2,426 (63.6%)	218 (50.9%)	< 0.001
Other Substance-Related Diagnosis, n (%)		2,848 (67.1%)	2,464 (64.6%)	384 (89.7%)	< 0.001
Outpatient Visit 6 Months Prior, n (%)					< 0.001
	No Prior Outpatient Visit	2,889 (68.1%)	2,803 (73.5%)	86 (20.1%)	
	Prior Outpatient Visit	1,353 (31.9%)	1,011 (26.5%)	342 (79.9%)	
MOUD Prescription 6 Months Prior, n (%)					< 0.001
	No MOUD	3,482 (82.1%)	3,390 (88.9%)	92 (21.5%)	
	Any MOUD	760 (17.9%)	424 (11.1%)	336 (78.5%)	
Overdose-Related Encounter, n (%)					< 0.001
	No overdose encounter	3,896 (91.8%)	3,481 (91.3%)	415 (97.0%)	
	Overdose encounter	346 (8.2%)	333 (8.7%)	13 (3.0%)	
Charlson Comorbidity Index, mean (SD)		3.2 (3.9)	3.3 (3.9)	2.1 (3.2)	< 0.001

### Sociodemographic and clinical determinants of MOUD prescription receipt during the index acute care encounter: adjusted logistic regression

Patients with a prior MOUD prescription within the six months preceding the acute care encounter had substantially higher odds of receiving MOUD during the index acute care encounter and remained the strongest independent predictor in adjusted analyses (aOR = 24.11, 95% CI [18.60, 31.53], *p* < 0.001). Similarly, patients with another substance-related diagnosis had higher odds of MOUD receipt compared with those without another substance-related diagnosis (aOR = 1.85, 95% CI [1.29, 2.69], *p* < 0.001). In contrast, having any documented mental health diagnosis was associated with lower odds of MOUD receipt during the acute care encounter (aOR = 0.73, 95% CI [0.57, 0.94], *p* = 0.02). Although overdose-related encounters were associated with lower adjusted odds of MOUD receipt (aOR = 0.56, 95% CI [0.29, 1.00], *p* = 0.06), the association did not reach conventional statistical significance after adjustment. Similarly, higher Charlson Comorbidity Index scores were associated with slightly lower odds of MOUD receipt, although the adjusted association was marginal (aOR = 0.96, 95% CI [0.92, 1.00], *p* = 0.05). Age group, sex, marital status, race/ethnicity, and insurance type were not significantly associated with MOUD receipt in adjusted analyses (Table 2). Model diagnostics demonstrated acceptable model performance and stability. The multivariable logistic regression model showed good discrimination, with an area under the receiver operating characteristic (ROC) curve of 0.884, and the Hosmer–Lemeshow goodness-of-fit test indicated no evidence of poor model fit (*p* = 0.09). Variance inflation factor (VIF) estimates suggested no evidence of problematic multicollinearity among included predictors (Supplementary File 3). Sensitivity analyses using alternative sample definitions and exclusion of overdose-related encounter from adjusted models yielded substantively similar findings, suggesting that the primary associations were robust across model specifications (Supplementary File 4).

**Table 2:** Bivariate and multivariable logistic regression models for MOUD receipt in patients with opioid-related diagnoses from southern california inpatient or emergency departments (*n* = 4,242)

Variable	Level	Unadjusted Model		Adjusted Model	
		Unadjusted Model (OR [95% CI])	*p*-value (Unadjusted)	Adjusted Model (aOR [95% CI])	*p*-value (Adjusted)
Age Group	18–34	Ref	-	Ref	-
	35–49	OR = 0.93 [0.72, 1.22]	0.62	aOR = 0.89 [0.64, 1.23]	0.47
	50–64	OR = 0.77 [0.58, 1.01]	0.06	aOR = 0.76 [0.53, 1.09]	0.14
	65+	OR = 0.57 [0.42, 0.77]	< 0.001	aOR = 0.73 [0.47, 1.13]	0.16
Sex	Male	Ref	-	Ref	-
	Female	OR = 0.89 [0.72, 1.09]	0.26	aOR = 1.10 [0.85, 1.42]	0.45
Marital Status	Single	Ref	-	Ref	-
	Married/Partnered	OR = 0.76 [0.59, 0.97]	0.03	aOR = 0.98 [0.72, 1.34]	0.91
	Previously Married/Other	OR = 0.57 [0.42, 0.77]	< 0.001	aOR = 0.92 [0.63, 1.33]	0.65
Race/Ethnicity	Non-Hispanic White	Ref	-	Ref	-
	Non-Hispanic Black	OR = 1.25 [0.91, 1.70]	0.16	aOR = 1.33 [0.90, 1.95]	0.15
	Hispanic/Latinx	OR = 1.00 [0.77, 1.28]	0.97	aOR = 0.84 [0.61, 1.14]	0.25
	Other/Mixed/Unknown	OR = 1.05 [0.77, 1.43]	0.74	aOR = 0.87 [0.60, 1.25]	0.46
Insurance Type	Commercial	Ref	-	Ref	-
	Medicare	OR = 0.60 [0.41, 0.86]	0.006	aOR = 0.90 [0.56, 1.42]	0.64
	Medicaid/Public	OR = 0.89 [0.64, 1.21]	0.46	aOR = 0.85 [0.57, 1.24]	0.41
	Self-Pay	OR = 1.16 [0.93, 1.46]	0.20	aOR = 1.05 [0.80, 1.38]	0.73
Any Mental Health Diagnosis	No	Ref	-	Ref	-
	Yes	OR = 0.59 [0.49, 0.73]	< 0.001	aOR = 0.73 [0.57, 0.94]	0.02
Other Substance-Related Diagnosis	No	Ref	-	Ref	-
	Yes	OR = 4.78 [3.52, 6.66]	< 0.001	aOR = 1.85 [1.29, 2.69]	< 0.001
MOUD Prescription 6 Months Prior	No MOUD	Ref	-	Ref	-
	Any MOUD	OR = 29.20 [22.80, 37.74]	< 0.001	aOR = 24.11 [18.60, 31.53]	< 0.001
Overdose-Related Encounter	No overdose encounter	Ref	-	Ref	-
	Overdose encounter	OR = 0.33 [0.18, 0.55]	< 0.001	aOR = 0.56 [0.29, 1.00]	0.06
Charlson Comorbidity Index	Per 1-point increase	OR = 0.91 [0.88, 0.94]	< 0.001	aOR = 0.96 [0.92, 1.00]	0.05

## Discussion

This study described the sociodemographic and clinical characteristics of patients from Southern California acute care settings with opioid-related involvement and estimated factors associated with MOUD receipt during acute care encounters. We found that having a prior MOUD prescription within six months preceding the index acute care encounter and having another substance-related diagnosis were associated with higher odds of receiving MOUD during acute care encounters. In contrast, patients with any mental health diagnosis had lower odds of MOUD receipt. Although opioid overdose–related encounters were associated with lower adjusted odds of MOUD receipt, this association did not reach conventional statistical significance after adjustment. Similarly, higher Charlson Comorbidity Index scores were associated with slightly lower odds of MOUD receipt, although this association was marginal. Findings from the primary analytic model were supported by sensitivity analyses, which demonstrated generally consistent directions of association across alternative sample definitions and model specifications. Overall, these findings suggest that MOUD receipt in acute care settings remains limited despite increasing recognition of acute care encounters as important opportunities for treatment initiation.

These findings should be interpreted within a population of patients with ORD care involvement who had an assigned primary care provider and utilized acute care services. As such, the results may not reflect patterns of MOUD receipt among all acute care patients, particularly those without established primary care. Notably, these findings highlight that even among patients with documented linkage to care, substantial gaps in MOUD initiation persist. The findings should also be interpreted within the context of California’s active statewide efforts to expand MOUD access, including the CA Bridge program and DHCS-led opioid response initiatives [18, 19]. Despite this supportive policy environment, MOUD receipt in our system remained limited, suggesting that the presence of state-level programs alone may not ensure consistent receipt within individual healthcare systems. Local implementation factors, including care coordination infrastructure and availability of specialized services such as addiction consultation teams, may play an important role in facilitating MOUD prescription. The absence of a formal addiction consultation service within our system may further contribute to variability in MOUD receipt, as such services are commonly integrated into acute care settings at many academic medical centers.

Patients prescribed MOUD within six months before the index acute care encounter had markedly higher odds of receiving MOUD during the acute care encounter compared with those without prior prescriptions, consistent with previous studies reporting increased likelihood of in-hospital MOUD receipt among patients with prior methadone or buprenorphine treatment [36–38]. This finding indicates that prior treatment engagements—specifically a previous MOUD prescription—facilitates MOUD receipt in acute care settings. Clinicians may preferentially offer MOUD to these patients because their established ORD, prior contact with addiction services, familiarity with prescribers, and documented treatment history suggest predictable adherence. Consequently, a prior MOUD prescription becomes a strong predictor of receiving MOUD during the acute care encounter, reflecting potential selection bias. These patterns underscore the need for MOUD protocols that ensure access for all patients with ORD, regardless of previous treatment engagement.

We also found that patients with any other SRDs had increased odds of receiving MOUD, contrasting earlier research reporting reduced MOUD receipt among patients with stimulant or polysubstance use [37, 39, 40]. Reduced receipt in earlier studies was attributed to provider hesitancy related to stigma toward stimulant co-use, uncertainties in managing overlapping withdrawal symptoms, and poor retention among patients who co-use methamphetamine [41, 42]. Additionally, abstinence-based program policies and functional motives reported by patients as reasons for stimulant use have historically contributed to lower MOUD receipt [43, 44]. Yet, some studies have reported higher MOUD receipt among patients with other psychoactive SRDs [45], consistent with our findings. This shift likely reflects evolving clinical practice, expanded addiction consultation services, changing provider attitudes toward polysubstance use, and policies promoting low-barrier, harm-reduction models that support MOUD initiation for patients with co-use of other specific SRDs who face elevated overdose risk [45, 46]. Our findings suggest a shift toward inclusive, harm-reduction MOUD practices, requiring further research to determine its persistence and impact on MOUD receipt for patients with ORDs and co-use of specific SRDs.

Unlike the unadjusted analyses, age was not independently associated with MOUD receipt after multivariable adjustment in the primary analytic model. Although unadjusted analyses suggested lower odds of MOUD receipt among older age groups, these associations attenuated after adjustment for clinical and treatment-related variables. This attenuation may indicate that factors such as prior treatment engagement, comorbidity burden, and co-occurring clinical conditions partially explain observed age-related differences in MOUD receipt. Nevertheless, prior research has documented lower MOUD utilization among older adults, including lower rates of buprenorphine receipt following opioid-related emergency department encounters [47], and reduced post-overdose MOUD prescribing among Medicare beneficiaries [4, 48]. Additional research is needed to better understand how age-related clinical complexity, healthcare access, insurance coverage, and provider decision-making influence MOUD receipt among older adults in acute care settings.

Although opioid overdose–related encounters were associated with lower adjusted odds of MOUD receipt, the association did not reach conventional statistical significance after adjustment. Nevertheless, the direction and magnitude of the association may still reflect clinically important gaps in MOUD initiation among patients presenting with overdose-related events. Prior research has consistently documented low rates of MOUD initiation following nonfatal overdose despite strong evidence that MOUD reduces recurrent overdose and mortality. National Medicare data show only 4.1% receive MOUD after nonfatal overdose [4]. In Massachusetts, fewer than one-third received MOUD after a non-fatal opioid overdose [49], and national insurance claims indicate only 1 in 6 received follow-up treatment within 90 days [50]. Our findings, together with earlier evidence, underscore a persistent treatment gap despite clear benefits of MOUD. The lack of statistical significance in the adjusted model may partially reflect limited statistical power due to the relatively small number of overdose-related encounters receiving MOUD in the study sample. Future research should focus on implementation strategies that increase MOUD uptake among patients managed for a non-fatal opioid overdose in the acute care setting.

Our results indicate that patients with any mental health diagnosis had lower odds of MOUD receipt during the index acute care encounter, consistent with prior studies. Research has shown that co-occurring mental health diagnoses reduce the likelihood of receiving MOUD, with the greatest gaps among patients with psychotic and mood disorders [51]. Another study has reported lower adherence to buprenorphine among patients with psychiatric comorbidities which may influence provider decisions to withhold MOUD in this population due to concerns about adherence, psychiatric symptom instability, or operational barriers [52, 53]. This treatment gap is concerning given the association between psychiatric comorbidity and elevated risk of non-fatal overdose and overdose mortality [3, 17, 54–56]. In 2022, 22% of overdose deaths involved a non–substance-related mental health disorder [57]. Given MOUD’s ability to reduce overdose and mortality [12], lower MOUD receipt may intensify risks of overdoses for patients with dual-diagnosis, making it essential for health systems and policymakers to prioritize closing access gaps in acute care settings.

### Strengths and limitations of the study

The present study has several strengths. First, using real-world electronic health records allowed examination of a critical outcome and facilitated population-level inferences for patients with ORDs. Second, reliance on ICD-10 codes reduced recall and social desirability bias. Third, multivariable logistic regression adjusting for key variables such as age, sex, race/ethnicity, mental health diagnoses, other substance-related diagnoses, overdose-related encounters, prior MOUD prescription, and medical comorbidity strengthened the ability to make inferences from our findings. Fourth, the study incorporated multiple sensitivity analyses using alternative opioid-related involvement definitions and model specifications, which demonstrated generally consistent directions of association and supported the robustness of the primary findings. Fifth, examining multiple issues experienced by patients, including SRDs, medical and mental health comorbidity, aligned with the real-world complexity of patients with ORDs and underscored the need for multidisciplinary collaboration and integration of addiction and mental health services.

However, several limitations warrant caution in the interpretation of our findings. First, coding inaccuracies may have led to misclassification of independent variables and the outcome. Second, unmeasured confounding may persist, as the study did not account for ORD severity. Third, the cross-sectional design utilized in this study means the temporal relationship between the independent variables and the outcome of interest is not assured, limiting causal inference. Fourth, selection bias in the acute care sample may limit generalizability to community populations with differing illness severity or care-seeking patterns. Fifth, the use of EHR data from a single academic health system (UC San Diego Health) and reliance on a specific registry may limit the generalizability of these findings to other healthcare settings, geographic regions, and populations with different care delivery structures and prescribing practices. Sixth, the broad definition of “opioid-related diagnosis involvement”—including an ORD ICD-10 code, prior or encounter MOUD prescription, or overdose encounter—may introduce heterogeneity in case identification. Although this approach improved case ascertainment, it remains a methodological limitation because the degree of overlap between these groups and the extent to which associations differed across criteria are unclear. Seventh, the relatively small number of overdose-related encounters receiving MOUD may have reduced statistical power and precision for estimates involving overdose-related encounter in adjusted analyses. To address this concern, variables were collapsed into broader clinically meaningful categories to improve model parsimony, and sensitivity analyses excluding overdose-related encounter from adjusted models were conducted. Findings from these sensitivity analyses were generally consistent with the primary model, suggesting that the overall pattern of associations was robust across model specifications. Eighth, restricting the sample to patients with a UC San Diego Health-assigned primary care provider (PCP) may further limit generalizability, as these individuals are more likely to be connected to care compared to patients without established primary care. This approach may also overrepresent patients with prior MOUD receipt. In addition, patients without an assigned PCP were excluded from the analytic dataset, and their demographic and clinical characteristics could not be compared, limiting assessment of potential selection bias. However, this restriction was necessary to ensure complete capture of outpatient prescribing and encounter data within the EHR, as care received outside the network is not available. Ninth, MOUD prescriptions obtained outside the health system—such as through stand-alone treatment programs—may not have been fully captured in the EHR. Although medication data were derived from multiple sources, including internal prescribing records, pharmacy-linked data, and patient-reported medication lists, some prior MOUD exposure may have been under-ascertained if not documented during clinical encounters. This limitation may have resulted in misclassification of prior treatment status and influenced observed associations. Tenth, having a UC San Diego Health PCP does not necessarily reflect active engagement in care, as many patients did not have recent outpatient visits. Therefore, PCP assignment should be interpreted as a proxy for potential access to care within the health system rather than actual healthcare utilization. Eleventh, excluding participants with sex recorded as “unknown” may also limit generalizability. Twelfth, the EHR lacked measures such as symptom severity, social support, patient perceptions of MOUD, and stigma, all of which are relevant to MOUD receipt. Thirteenth, the study did not include geographic-level variables, such as rural–urban residence, because county-level identifiers were unavailable in the dataset. Rural–urban differences are important factors influencing access to MOUD and may contribute to variation in treatment receipt. Future research should incorporate geographic classification measures, such as Rural–Urban Continuum Codes (RUCC), to better understand the role of location in MOUD access and utilization. Fourteenth, although social determinants of health are important influences on MOUD receipt, these measures were not reliably captured in the dataset. Due to substantial missingness and under-documentation, the SDOH variable was excluded from the final analysis. Despite these limitations, the study highlights a critical clinical and policy-relevant gap contributing to morbidity and mortality among patients with ORDs.

### Clinical, policy, and research implications of the study

Clinical, policy, and research implications emerge from our findings. Clinically, there is a need to expand addiction treatment within acute care settings and reduce reliance on referrals when MOUD initiation can occur during the encounter. ED-initiated buprenorphine improves treatment engagement compared with brief intervention and referral [58]. Health systems should strengthen interdisciplinary addiction consultation services (ACS)—including addiction medicine, psychiatry, social work, and peer navigators—which have been shown to increase MOUD initiation and post-discharge engagement [59]. From a policy standpoint, structural, regulatory, and financing reforms are needed to integrate addiction treatment into acute care so that MOUD and behavioral health services become routine components of care. Although ED-initiated MOUD reduces overdose and mortality, many hospitals still lack formal programs due to regulatory uncertainty, reimbursement challenges, and workforce shortages [60, 61]. Expanding payment models to support ACS, funding to cover medications for uninsured, peer navigators, and co-located behavioral health services may address these barriers [62–64]. The persistence of low MOUD receipt despite California’s active statewide opioid response initiatives also suggests that policy expansion alone may not ensure consistent implementation across healthcare systems, highlighting the importance of local infrastructure, provider training, and operational integration of addiction services. Future research should include broader ED populations, including those without established primary care to complement our findings and evaluate implementation strategies—such as peer recovery models—to improve MOUD receipt [65]. Additional studies are needed to elucidate mechanisms linking mental health comorbidity to lower MOUD initiation and to assess alternative induction approaches, including micro-induction and extended-release formulations, for patients with complex psychiatric profiles [66, 67]. Future research should also further evaluate factors associated with lower MOUD receipt among patients presenting with overdose-related encounters, including potential barriers related to care transitions, stigma, clinical complexity, and availability of addiction consultation services. Longitudinal research is also warranted to clarify temporal relationships among social determinants of health, medical comorbidity, mental health comorbidity, and MOUD receipt, thereby informing targeted interventions for populations most affected by the opioid epidemic in the United States. Future studies should also incorporate geographic-level variables, such as rural–urban residence (e.g., RUCC codes), to better understand how location influences access to and utilization of MOUD.

## Conclusion

This study examined sociodemographic and clinical factors associated with MOUD receipt in acute care settings among patients with ORD care involvement who had an assigned primary care provider and utilized acute care services. Findings from the primary analysis showed that prior MOUD prescription within six months preceding the index acute care encounter was strongly associated with MOUD receipt during the acute care encounter, indicating that prior treatment engagement facilitates continued MOUD receipt in acute care settings. This finding should be interpreted in the context of the study population, as restricting the sample to patients with an assigned primary care provider may overrepresent individuals with prior treatment exposure and limit generalizability to all acute care patients. This also suggests potential selection bias, underscoring the need for inclusive addiction care that offers MOUD to all patients regardless of treatment history.

Having any other SRDs was associated with higher odds of MOUD receipt, suggesting a shift toward more inclusive, harm-reduction–oriented practices for individuals with co-occurring or polysubstance use who are at elevated risk of morbidity and mortality. Further research is needed to determine whether this pattern is sustained across other health systems and translates into increased MOUD receipt. Unlike some prior studies, age was not independently associated with MOUD receipt after adjustment. Although unadjusted analyses suggested lower odds of MOUD receipt among older adults, these associations attenuated after accounting for clinical and treatment-related factors. Additional research is needed to better understand the role of healthcare access, treatment engagement, comorbidity burden, and insurance coverage in shaping MOUD receipt among older adults in acute care settings.

Although opioid overdose–related encounters were associated with lower adjusted odds of MOUD receipt, the association did not reach conventional statistical significance after adjustment. Nevertheless, the direction and magnitude of the association may still reflect important missed opportunities for MOUD initiation among patients presenting with overdose-related events. Additional research is needed to identify barriers to MOUD initiation following nonfatal overdose and to evaluate implementation strategies that improve treatment uptake for this high-risk population.

Finally, individuals with mental health diagnoses had lower odds of receiving MOUD, suggesting that clinical complexity, drug–drug interaction concerns, and anticipated disengagement may influence prescribing. Integrated care models and research on tailored induction approaches, including micro-induction and extended-release formulations, are urgently needed to improve MOUD receipt for patients with co-occurring mental health conditions and reduce the morbidity and mortality experienced by this population. Overall, these findings highlight persistent gaps in MOUD receipt even among patients with established connections to care, underscoring the need for system-level strategies to improve initiation of evidence-based treatment in acute care settings.

## Supplementary Information

Below is the link to the electronic supplementary material.

Supplementary Material 1

Supplementary Material 2

Supplementary Material 3

Supplementary Material 4

## Data Availability

The data set used to conduct the analysis in this study is not published publicly to protect the privacy of the patients. If researchers are interested in such data, they are free to contact the corresponding author for details on data access procedures given appropriate IRB approval.

## Abbreviations

SRD: Substance-related diagnosis
SRDs: Substance-related diagnoses
ORD: Opioid-related Diagnosis
MOUD: Medication for Opioid Use Disorder
IP: In-patient
ED: Emergency department
HIPAA: Health Insurance Portability and Accountability Act
IRB: Kuali Institutional Review Board (IRB)
EHR: Electronic Health Records
