# Supplementary material for: A missed opportunity: receipt of medication for opioid use disorder in Southern California acute care settings
Source: Addict Sci Clin Pract. 2026 Jul 16;21:73. doi: 10.1186/s13722-026-00703-0 (PMC13660156; doi:10.1186/s13722-026-00703-0)
Supplement: Supplementary file 1 [file 13722_2026_703_MOESM1_ESM.docx]

**Supplemental Material 1**

**Table A: International Classification of Diseases, Tenth Revision, Clinical Modification (ICD-10) codes for substance-related diagnoses, and overdose-related diagnosis**

| **Condition/Diagnosis** | **ICD-10 Codes** |
| --- | --- |
| **Any Substance-related diagnosis (SRD)*** |  |
| Alcohol-related diagnosis | F10.xx |
| Opioid-related diagnosis | F11.xx |
| Cannabis-related diagnosis | F12.xx |
| Sedative, hypnotic, or anxiolytic-related diagnosis | F13.xx |
| Cocaine-related diagnosis | F14.xx |
| Other stimulant-related diagnosis | F15.xx |
| Hallucinogen-related diagnosis | F16.xx |
| Nicotine-related diagnosis | F17.xx |
| Inhalant-related diagnosis | F18.xx |
| Other psychoactive substance-related diagnosis | F19.xx |
| **Other Specific Substance Related Diagnosis** |  |
| Alcohol-related diagnosis | F10.xx |
| Cannabis-related diagnosis | F12.xx |
| Sedative, hypnotic, or anxiolytic-related diagnosis | F13.xx |
| Cocaine-related diagnosis | F14.xx |
| Other stimulant-related diagnosis | F15.xx |
| Hallucinogen-related diagnosis | F16.xx |
| Nicotine-related diagnosis | F17.xx |
| Inhalant-related diagnosis | F18.xx |
| Other psychoactive substance-related diagnosis | F19.xx |
| **Overdose-related diagnosis** |  |
| Alcohol-Related Overdose | T51.0-T51.0X4S |
| Opioid-Related Overdose | T40.0-T40.496S, T40.6-T40.696S |
| Cannabis-Related Overdose | T40.7-T40.7X6S |
| Sedative, hypnotic, or anxiolytics related overdose | T42.3-T42,4X6S |
| Stimulant-Related Overdose | T40.5-T40.5X6S, T43.6-T43.696S |
| Hallucinogen-Related Overdose | T40.8-T40.996S |

Note: *The ICD-10 F code categories of F1X.xx include substance intoxication, substance withdrawal, substance use, and substance use disorder diagnosis codes

**Table B: International Classification of Diseases, Tenth Revision, Clinical Modification (ICD-10) codes Accounted for in the Computation of the Charlson Comorbidity Index**

| **Condition/Diagnosis** | **ICD-10 Codes** |
| --- | --- |
| Viral Hepatitis | B15.x-B19.x |
| AIDS/HIV | B20.x - B22.x, B24.x |
| Myocardial infarction | I21.x, I22.x, I25.2 |
| Congestive heart failure | I09.9, I11.0, I13.0, I13.2, I25.5, I42.0, I42.5 - I42.9, I43.x, I50.x, P29.0 |
| Peripheral vascular disease | I70.x, I71.x, I73.1, I73.8, I73.9, I77.1, I79.0, I79.2, K55.1, K55.8, K55.9, Z95.8, Z95.9 |
| Cerebrovascular disease | G45.x, G46.x, H34.0, I60.x - I69.x |
| Dementia | F00.x - F03.x, F05.1, G30.x, G31.1 |
| Chronic pulmonary disease | I27.8, I27.9, J40.x - J47.x, J60.x - J67.x, J68.4, J70.1, J70.3 |
| Rheumatic disease | M05.x, M06.x, M31.5, M32.x - M34.x, M35.1, M35.3, M36.0 |
| Peptic ulcer disease | K25.x - K28.x |
| Mild liver disease | B18.x, K70.0 - K70.3, K70.9, K71.3 - K71.5, K71.7, K73.x, K74.x, K76.0, K76.2 - K76.4, K76.8, K76.9, Z94.4 |
| Diabetes without chronic complication | E10.0, E10.1, E10.6, E10.8, E10.9, E11.0, E11.1, E11.6, E11.8, E11.9, E12.0, E12.1, E12.6, E12.8, E12.9, E13.0, E13.1, E13.6, E13.8, E13.9, E14.0, E14.1, E14.6, E14.8, E14.9 |
| Diabetes with chronic complication | E10.2 - E10.5, E10.7, E11.2 - E11.5, E11.7, E12.2 - E12.5, E12.7, E13.2 - E13.5, E13.7, E14.2 - E14.5, E14.7 |
| Hemiplegia or paraplegia | G04.1, G11.4, G80.1, G80.2, G81.x, G82.x, G83.0 - G83.4, G83.9 |
| Renal disease | I12.0, I13.1, N03.2 - N03.7, N05.2 - N05.7, N18.x, N19.x, N25.0, Z49.0 - Z49.2, Z94.0, Z99.2 |
| Any malignancy (except skin) | C00.x - C26.x, C30.x - C34.x, C37.x - C41.x, C43.x, C45.x - C58.x, C60.x - C76.x, C81.x - C85.x, C88.x, C90.x - C97.x |
| Moderate or severe liver disease | I85.0, I85.9, I86.4, I98.2, K70.4, K71.1, K72.1, K72.9, K76.5, K76.6, K76.7 |
| Metastatic solid tumour | C77.x - C80.x |

**Table C: International Classification of Diseases, Tenth Revision, Clinical Modification (ICD-10) codes for serious mental illness, and non-serious mental illness used in the variable any mental health diagnosis**

| **Condition/Diagnosis** | **ICD-10 Codes** |
| --- | --- |
| **Severe Mental Health Diagnosis** |  |
| Schizophrenia | F20.xx |
| Schizotypal disorder | F21.xx |
| Persistent delusional disorder | F22.xx |
| Schizoaffective disorder | F25.xx |
| Manic episode | F30.xx |
| Bipolar disorder | F31.xx |
| Major depressive symptom severe | F32.2-F32.3, F33.2-F33.2 |
| **Non-Severe Mental Health Diagnosis** |  |
| Delusional disorders | Delusional disorders F22 |
| Brief psychotic disorders | Brief psychotic disorders F23 |
| Other psychotic disorder not due to a substance or known physiologic condition | F28 |
| Unspecified psychosis | F29 |
| Major depressive disorder mild or moderate | F32.0-F32.1, F32.4-F32.9, F33.0-F33.1, F33.4-F33.9 |
| Persistent mood disorder | F39 |
| Reaction to severe stress, and adjustment disorders (includes post-traumatic stress syndrome (PTSD)) | F43.xx |
| Obsessive compulsive disorder | F42.xx |
| Phobic anxiety disorder | F40.xx |
| Other anxiety disorder | F41.xx |
| Eating disorder | F50.xx |
| Specific personality disorder | F60.xx |
| Impulse disorder | F63.xx |
