# Supplementary material for: A missed opportunity: receipt of medication for opioid use disorder in Southern California acute care settings
Source: Addict Sci Clin Pract. 2026 Jul 16;21:73. doi: 10.1186/s13722-026-00703-0 (PMC13660156; doi:10.1186/s13722-026-00703-0)
Supplement: Supplementary file 2 [file 13722_2026_703_MOESM2_ESM.docx]

**Supplemental Material 2. Alternative Sample Definitions Used in Primary and Sensitivity Analyses**

| **Model** | **Sample Definition** | **Description** | **Sample Size (N)** | **Purpose** |
| --- | --- | --- | --- | --- |
| Primary Model (Model 1) | Opioid-related diagnosis (ORD) and/or prior MOUD prescription, MOUD during acute care encounter, or overdose-related encounter | Broad opioid-related involvement definition incorporating diagnostic, treatment, and overdose-related indicators | 4,242 | Primary analytic model |
| Sensitivity Model 2 | Primary model excluding overdose-related encounter from adjusted analyses | Broad opioid-related involvement definition with overdose-related encounter removed from multivariable models | 4,242 | Sensitivity analysis assessing the influence of sparse cells associated with overdose-related encounters |
| Sensitivity Model 3 | ORD and/or MOUD during acute care encounter or overdose-related encounter3 | Alternative opioid-related involvement definition excluding patients identified solely through prior MOUD prescription | 3,919 | Sensitivity analysis evaluating robustness to exclusion of prior MOUD-only inclusion |
| Sensitivity Model 4 | Alternative opioid-related involvement definition excluding overdose-related encounter from adjusted analyses | ORD and/or MOUD during acute care encounter or overdose-related encounter definition with overdose-related encounter removed from adjusted analyses | 3,919 | Sensitivity analysis assessing robustness of findings after removal of overdose-related encounter |
| Sensitivity Model 5 | ORD and/or overdose-related encounter documented in the EHR | Restrictive EHR-based opioid-related involvement definition limited to diagnostic and overdose-related indicators | 3,585 | Sensitivity analysis evaluating robustness under a narrower diagnosis-based definition |
| Sensitivity Model 6 | Restrictive EHR-based opioid-related involvement definition excluding overdose-related encounter from adjusted analyses | ORD and/or overdose-related encounter documented in the EHR definition with overdose-related encounter removed from adjusted analyses | 3,585 | Sensitivity analysis assessing robustness under restrictive diagnostic definitions after removal of overdose-related encounter |

**Abbreviations: MOUD = medications for opioid use disorder; ORD = opioid-related diagnosis; EHR = electronic health record.**
