# Supplementary material for: A missed opportunity: receipt of medication for opioid use disorder in Southern California acute care settings
Source: Addict Sci Clin Pract. 2026 Jul 16;21:73. doi: 10.1186/s13722-026-00703-0 (PMC13660156; doi:10.1186/s13722-026-00703-0)
Supplement: Supplementary file 3 [file 13722_2026_703_MOESM3_ESM.docx]

**Model 1 ( Diagnostics for Final MOUD Logistic Regression Model, N = 4,242)**

The area under the ROC curve (AUC) for the final multivariable logistic regression model was 0.884, indicating good discrimination. The Hosmer-Lemeshow goodness-of-fit test yielded a p-value of 0.09, indicating no evidence of poor model fit.

## 1.1. Variance Inflation Factor

| **Variable** | **GVIF** | **Df** | **Adjusted VIF** |
| --- | --- | --- | --- |
| Age Group | 1.66 | 3 | 1.09 |
| SEX | 1.09 | 1 | 1.04 |
| Marital Status | 1.23 | 2 | 1.05 |
| Race/Ethnicity | 1.10 | 3 | 1.02 |
| Insurance Type | 1.32 | 3 | 1.05 |
| Any Menta Health Diagnosis | 1.10 | 1 | 1.05 |
| Other Substance Specific SRDs New | 1.10 | 1 | 1.05 |
| MOUD Within 6 Months Prior | 1.08 | 1 | 1.04 |
| Overdose-related encounter | 1.02 | 1 | 1.01 |
| Charlson Comorbidity Index | 1.26 | 1 | 1.12 |

## 1.2. Hosmer-Lemeshow Goodness-of-Fit Test

| **Test** | **Statistic** | **DF** | **P_value** |
| --- | --- | --- | --- |
| Hosmer-Lemeshow | 13.61 | 8 | 0.09 |

## 1.3. Model Discrimination

| **Statistic** | **AUC** |
| --- | --- |
| Area under the ROC curve | 0.884 |

## 1.4. Calibration by Risk Decile

| **Decile** | **Mean predicted** | **Observed rate** | **n** |
| --- | --- | --- | --- |
| 1 | 0.011 | 0.002 | 425 |
| 2 | 0.015 | 0.012 | 425 |
| 3 | 0.019 | 0.014 | 424 |
| 4 | 0.023 | 0.019 | 424 |
| 5 | 0.026 | 0.021 | 424 |
| 6 | 0.030 | 0.024 | 424 |
| 7 | 0.036 | 0.031 | 424 |
| 8 | 0.045 | 0.066 | 424 |
| 9 | 0.295 | 0.333 | 424 |
| 10 | 0.510 | 0.488 | 424 |

## 1.5. Figure 1. ROC Curve for Final MOUD Model

## 1.6. Figure 2. Calibration Plot for Final MOUD Model

**Model 2 (Diagnostics for MOUD Logistic Regression Model Excluding Overdose-Related Encounter,** **N = 4,242 )**

The area under the ROC curve (AUC) for the multivariable logistic regression model excluding overdose-related encounter was 0.883, indicating good discrimination.

The Hosmer-Lemeshow goodness-of-fit test yielded a p-value of 0.08, indicating no evidence of poor model fit.

1.7. **Variance Inflation Factor**

| **Variable** | **GVIF** | **Df** | **Adjusted_VIF** |
| --- | --- | --- | --- |
| Age Group | 1.66 | 3 | 1.09 |
| SEX | 1.09 | 1 | 1.04 |
| Marital Status | 1.23 | 2 | 1.05 |
| Race/Ethnicity | 1.10 | 3 | 1.02 |
| Insurance Type | 1.32 | 3 | 1.05 |
| Any Mental Health Diagnosis | 1.10 | 1 | 1.05 |
| Other Substance Specific SRDs | 1.10 | 1 | 1.05 |
| MOUD within 6 months prior | 1.07 | 1 | 1.04 |
| Charlson Comorbidity Index | 1.26 | 1 | 1.12 |

1.8. **Hosmer-Lemeshow Goodness-of-Fit Test**

| **Test** | **Statistic** | **DF** | **P_value** |
| --- | --- | --- | --- |
| Hosmer-Lemeshow | 14.08 | 8 | 0.08 |

1.9. **Model Discrimination**

| **Statistic** | **AUC** |
| --- | --- |
| Area under the ROC curve | 0.883 |

1.10. **Calibration by Risk Decile**

| **Decile** | **Mean Predicted** | **Observed rate** | **n** |
| --- | --- | --- | --- |
| 1 | 0.011 | 0.002 | 425 |
| 2 | 0.015 | 0.014 | 425 |
| 3 | 0.019 | 0.009 | 424 |
| 4 | 0.023 | 0.019 | 424 |
| 5 | 0.026 | 0.021 | 424 |
| 6 | 0.031 | 0.031 | 424 |
| 7 | 0.036 | 0.028 | 424 |
| 8 | 0.044 | 0.068 | 424 |
| 9 | 0.298 | 0.323 | 424 |
| 10 | 0.507 | 0.493 | 424 |

1.11. **Figure 1. ROC Curve for MOUD Model Excluding Overdose-Related Encounter**

1.12. **Figure 2. Calibration Plot for MOUD Model Excluding Overdose-Related Encounter**

**Model 3 ( Diagnostics for Final MOUD Logistic Regression Model, N = 3,919)**

The area under the ROC curve (AUC) for the final multivariable logistic regression model was 0.935, indicating good discrimination. The Hosmer-Lemeshow goodness-of-fit test yielded a p-value of 0.09, indicating no evidence of poor model fit.

## 1.13. Variance Inflation Factor

| **Variable** | **GVIF** | **Df** | **Adjusted_VIF** |
| --- | --- | --- | --- |
| Age Group | 1.69 | 3 | 1.09 |
| SEX | 1.09 | 1 | 1.05 |
| Marital Status | 1.28 | 2 | 1.06 |
| Race/Ethnicity | 1.12 | 3 | 1.02 |
| Insurance Type | 1.32 | 3 | 1.05 |
| Any Mental Health Diagnosis | 1.14 | 1 | 1.07 |
| Other Substance Specific SRDs | 1.10 | 1 | 1.05 |
| MOUD within 6 months prior | 1.13 | 1 | 1.06 |
| Overdose-related encounter | 1.02 | 1 | 1.01 |
| Charlson Comorbidity Index | 1.29 | 1 | 1.14 |

## 1.14. Hosmer-Lemeshow Goodness-of-Fit Test

| **Test** | **Statistic** | **DF** | **P_value** |
| --- | --- | --- | --- |
| Hosmer-Lemeshow | 13.87 | 8 | 0.09 |

## 1.15. Model Discrimination

| **Statistic** | **AUC** |
| --- | --- |
| Area under the ROC curve | 0.935 |

## 1.16. Calibration by Risk Decile

| **Decile** | **Mean predicted** | **Observed rate** | **n** |
| --- | --- | --- | --- |
| 1 | 0.003 | 0.003 | 392 |
| 2 | 0.006 | 0.010 | 392 |
| 3 | 0.010 | 0.013 | 392 |
| 4 | 0.015 | 0.010 | 392 |
| 5 | 0.021 | 0.026 | 392 |
| 6 | 0.027 | 0.013 | 392 |
| 7 | 0.033 | 0.023 | 392 |
| 8 | 0.050 | 0.043 | 392 |
| 9 | 0.112 | 0.148 | 392 |
| 10 | 0.818 | 0.806 | 391 |

## 1.17. Figure 1. ROC Curve for Final MOUD Model

## 1.18. Figure 2. Calibration Plot for Final MOUD Model

**Model 4 (Diagnostics for MOUD Logistic Regression Model Excluding Overdose-Related Encounter, N = 3,919)**

The area under the ROC curve (AUC) for the multivariable logistic regression model excluding overdose-related encounter was 0.933, indicating good discrimination.

The Hosmer-Lemeshow goodness-of-fit test yielded a p-value of 0.18, indicating no evidence of poor model fit.

## 1.19. Variance Inflation Factor

| **Variable** | **GVIF** | **Df** | **Adjusted VIF** |
| --- | --- | --- | --- |
| Age Group | 1.67 | 3 | 1.09 |
| SEX | 1.09 | 1 | 1.04 |
| Marital Status | 1.27 | 2 | 1.06 |
| Race/Ethnicity | 1.12 | 3 | 1.02 |
| Insurance Type | 1.32 | 3 | 1.05 |
| Any Mental Health Diagnosis | 1.14 | 1 | 1.07 |
| Other Substance Specific SRDs | 1.10 | 1 | 1.05 |
| MOUD within 6 months prior | 1.12 | 1 | 1.06 |
| Charlson Comorbidity Index | 1.29 | 1 | 1.14 |

## 1.20. Hosmer-Lemeshow Goodness-of-Fit Test

| **Test** | **Statistic** | **DF** | **P_value** |
| --- | --- | --- | --- |
| Hosmer-Lemeshow | 11.44 | 8 | 0.18 |

## 1.21. Model Discrimination

| **Statistic** | **AUC** |
| --- | --- |
| Area under the ROC curve | 0.933 |

## 1.22. Calibration by Risk Decile

| **Decile** | **Mean predicted** | **Observed rate** | **n** |
| --- | --- | --- | --- |
| 1 | 0.004 | 0.005 | 392 |
| 2 | 0.006 | 0.013 | 392 |
| 3 | 0.011 | 0.008 | 392 |
| 4 | 0.016 | 0.013 | 392 |
| 5 | 0.021 | 0.020 | 392 |
| 6 | 0.027 | 0.018 | 392 |
| 7 | 0.033 | 0.023 | 392 |
| 8 | 0.051 | 0.048 | 392 |
| 9 | 0.109 | 0.143 | 392 |
| 10 | 0.817 | 0.803 | 391 |

## 1.23. Figure 1. ROC Curve for MOUD Model Excluding Overdose-Related Encounter

## 1.24. Figure 2. Calibration Plot for MOUD Model Excluding Overdose-Related Encounter

**Model 5 (Diagnostics for Final MOUD Logistic Regression Model, N=3,585)**

The area under the ROC curve (AUC) for the final multivariable logistic regression model was 0.889, indicating good discrimination. The Hosmer-Lemeshow goodness-of-fit test yielded a p-value of 0.07, indicating no evidence of poor model fit.

## 1.25. Variance Inflation Factor

| **Variable** | **GVIF** | **Df** | **Adjusted VIF** |
| --- | --- | --- | --- |
| Age Group | 1.92 | 3 | 1.11 |
| SEX | 1.15 | 1 | 1.07 |
| Marital Status | 1.47 | 2 | 1.10 |
| Race/Ethnicity | 1.15 | 3 | 1.02 |
| Insurance Type | 1.53 | 3 | 1.07 |
| Any Mental Health Diagnosis | 1.13 | 1 | 1.06 |
| Other Substance Specific SRDs | 1.17 | 1 | 1.08 |
| MOUD within 6 months prior | 1.04 | 1 | 1.02 |
| Overdose-related encounter | 1.03 | 1 | 1.02 |
| Charlson Comorbidity Index | 1.36 | 1 | 1.17 |

## 1.26. Hosmer-Lemeshow Goodness-of-Fit Test

| **Test** | **Statistic** | **DF** | **P_value** |
| --- | --- | --- | --- |
| Hosmer-Lemeshow | 14.54 | 8 | 0.07 |

## 1.27. Model Discrimination

| **Statistic** | **AUC** |
| --- | --- |
| Area under the ROC curve | 0.889 |

## 1.28. Calibration by Risk Decile

| **Decile** | **Mean predicted** | **Observed rate** | **n** |
| --- | --- | --- | --- |
| 1 | 0.003 | 0.006 | 359 |
| 2 | 0.004 | 0.006 | 359 |
| 3 | 0.004 | 0.008 | 359 |
| 4 | 0.004 | 0.003 | 359 |
| 5 | 0.005 | 0.003 | 359 |
| 6 | 0.006 | 0.014 | 358 |
| 7 | 0.006 | 0.000 | 358 |
| 8 | 0.007 | 0.000 | 358 |
| 9 | 0.008 | 0.003 | 358 |
| 10 | 0.215 | 0.221 | 358 |

## 1.29. Figure 1. ROC Curve for Final MOUD Model

## 1.30. Figure 2. Calibration Plot for Final MOUD Model

**Model 6 ( Diagnostics for MOUD Logistic Regression Model Excluding Overdose-Related Encounter, N=3,585)**

The area under the ROC curve (AUC) for the multivariable logistic regression model excluding overdose-related encounter was 0.891, indicating good discrimination.

The Hosmer-Lemeshow goodness-of-fit test yielded a p-value of 0.54, indicating no evidence of poor model fit.

## 1.31. Variance Inflation Factor

| **Variable** | **GVIF** | **Df** | **Adjusted_VIF** |
| --- | --- | --- | --- |
| Age Group | 1.91 | 3 | 1.11 |
| SEX | 1.15 | 1 | 1.07 |
| Marital Status | 1.46 | 2 | 1.10 |
| Race/Ethnicity | 1.15 | 3 | 1.02 |
| Insurance | 1.51 | 3 | 1.07 |
| Any Menta Health Diagnosis | 1.13 | 1 | 1.06 |
| Other Substance Specific SRDs | 1.17 | 1 | 1.08 |
| MOUD within 6 months prior | 1.04 | 1 | 1.02 |
| Charlson Comorbidity Index | 1.36 | 1 | 1.17 |

## 1.32. Hosmer-Lemeshow Goodness-of-Fit Test

| **Test** | **Statistic** | **DF** | **P_value** |
| --- | --- | --- | --- |
| Hosmer-Lemeshow | 6.98 | 8 | 0.54 |

## 1.33. Model Discrimination

| **Statistic** | **AUC** |
| --- | --- |
| Area under the ROC curve | 0.891 |

## 1.34. Calibration by Risk Decile

| **Decile** | **Mean predicted** | **Observed rate** | **n** |
| --- | --- | --- | --- |
| 1 | 0.003 | 0.006 | 359 |
| 2 | 0.004 | 0.006 | 359 |
| 3 | 0.004 | 0.006 | 359 |
| 4 | 0.004 | 0.008 | 359 |
| 5 | 0.005 | 0.003 | 359 |
| 6 | 0.006 | 0.008 | 358 |
| 7 | 0.006 | 0.000 | 358 |
| 8 | 0.007 | 0.003 | 358 |
| 9 | 0.008 | 0.008 | 358 |
| 10 | 0.215 | 0.215 | 358 |

## 1.35. Figure 1. ROC Curve for MOUD Model Excluding Overdose-Related Encounter

## 1.36. Figure 2. Calibration Plot for MOUD Model Excluding Overdose-Related Encounter
