# Supplementary material for: A missed opportunity: receipt of medication for opioid use disorder in Southern California acute care settings
Source: Addict Sci Clin Pract. 2026 Jul 16;21:73. doi: 10.1186/s13722-026-00703-0 (PMC13660156; doi:10.1186/s13722-026-00703-0)
Supplement: Supplementary file 4 [file 13722_2026_703_MOESM4_ESM.docx]

# Supplementary Table S1. Sensitivity Analysis: Multivariable Logistic Regression Model Excluding Overdose-Related Encounter Among Patients With Opioid-Related Diagnoses, Prior MOUD Prescription, MOUD During Acute Care Encounter, or Overdose-Related Encounter (N = 4,242)

|  |  | **Unadjusted Model** |  | **Adjusted Model** |  |
| --- | --- | --- | --- | --- | --- |
| **Variable** | **Level** | **Unadjusted Model (OR [95% CI])** | **p-value (Unadjusted)** | **Adjusted Model (aOR [95% CI])** | **p-value (Adjusted)** |
| Age Group | 18-34 | Ref | - | Ref | - |
|  | 35-49 | OR = 0.93 [0.72, 1.22] | 0.62 | aOR = 0.89 [0.65, 1.24] | 0.50 |
|  | 50-64 | OR = 0.77 [0.58, 1.01] | 0.06 | aOR = 0.77 [0.54, 1.11] | 0.16 |
|  | 65+ | OR = 0.57 [0.42, 0.77] | <0.001 | aOR = 0.74 [0.47, 1.15] | 0.18 |
| Sex | Male | Ref | - | Ref | - |
|  | Female | OR = 0.89 [0.72, 1.09] | 0.26 | aOR = 1.12 [0.87, 1.44] | 0.39 |
| Marital Status | Single | Ref | - | Ref | - |
|  | Married/Partnered | OR = 0.76 [0.59, 0.97] | 0.03 | aOR = 0.99 [0.72, 1.35] | 0.96 |
|  | Previously Married/Other | OR = 0.57 [0.42, 0.77] | <0.001 | aOR = 0.91 [0.62, 1.31] | 0.61 |
| Race/Ethnicity | Non-Hispanic White | Ref | - | Ref | - |
|  | Non-Hispanic Black | OR = 1.25 [0.91, 1.70] | 0.16 | aOR = 1.33 [0.90, 1.95] | 0.15 |
|  | Hispanic/Latinx | OR = 1.00 [0.77, 1.28] | 0.97 | aOR = 0.83 [0.61, 1.13] | 0.24 |
|  | Other/Mixed/Unknown | OR = 1.05 [0.77, 1.43] | 0.74 | aOR = 0.87 [0.60, 1.25] | 0.46 |
| Insurance Type | Commercial | Ref | - | Ref | - |
|  | Medicare | OR = 0.60 [0.41, 0.86] | 0.006 | aOR = 0.90 [0.56, 1.43] | 0.67 |
|  | Medicaid/Public | OR = 0.89 [0.64, 1.21] | 0.46 | aOR = 0.83 [0.56, 1.22] | 0.35 |
|  | Self-Pay | OR = 1.16 [0.93, 1.46] | 0.20 | aOR = 1.04 [0.79, 1.37] | 0.76 |
| Any Mental Health Diagnosis | No | Ref | - | Ref | - |
|  | Yes | OR = 0.59 [0.49, 0.73] | <0.001 | aOR = 0.73 [0.57, 0.94] | 0.02 |
| Other Substance-Related Diagnosis | No | Ref | - | Ref | - |
|  | Yes | OR = 4.78 [3.52, 6.66] | <0.001 | aOR = 1.88 [1.32, 2.73] | <0.001 |
| MOUD Prescription 6 Months Prior | No MOUD | Ref | - | Ref | - |
|  | Any MOUD | OR = 29.20 [22.80, 37.74] | <0.001 | aOR = 24.70 [19.07, 32.27] | <0.001 |
| Charlson Comorbidity Index | Per 1-point increase | OR = 0.91 [0.88, 0.94] | <0.001 | aOR = 0.96 [0.92, 1.00] | 0.06 |

Abbreviations: OR = odds ratio; aOR = adjusted odds ratio; CI = confidence interval.

# Supplementary Table S2. Sensitivity Analysis: Logistic Regression Model Using Alternative Opioid-Related Involvement Definition Among Patients With Opioid-Related Diagnoses and/or MOUD During Acute Care Encounter or Overdose-Related Encounter (N = 3,919)

|  |  | **Unadjusted Model** |  | **Adjusted Model** |  |
| --- | --- | --- | --- | --- | --- |
| **Variable** | **Level** | **Unadjusted Model (OR [95% CI])** | **p-value (Unadjusted)** | **Adjusted Model (aOR [95% CI])** | **p-value (Adjusted)** |
| Age Group | 18-34 | Ref | - | Ref | - |
|  | 35-49 | OR = 0.95 [0.73, 1.25] | 0.73 | aOR = 1.04 [0.67, 1.61] | 0.86 |
|  | 50-64 | OR = 0.78 [0.59, 1.03] | 0.08 | aOR = 1.04 [0.64, 1.69] | 0.86 |
|  | 65+ | OR = 0.57 [0.42, 0.77] | <0.001 | aOR = 1.07 [0.59, 1.93] | 0.82 |
| Sex | Male | Ref | - | Ref | - |
|  | Female | OR = 0.87 [0.70, 1.06] | 0.17 | aOR = 1.17 [0.83, 1.64] | 0.37 |
| Marital Status | Single | Ref | - | Ref | - |
|  | Married/Partnered | OR = 0.75 [0.58, 0.95] | 0.02 | aOR = 1.07 [0.70, 1.62] | 0.75 |
|  | Previously Married/Other | OR = 0.55 [0.40, 0.73] | <0.001 | aOR = 0.77 [0.47, 1.25] | 0.30 |
| Race/Ethnicity | Non-Hispanic White | Ref | - | Ref | - |
|  | Non-Hispanic Black | OR = 1.26 [0.91, 1.71] | 0.15 | aOR = 1.73 [1.04, 2.83] | 0.03 |
|  | Hispanic/Latinx | OR = 1.02 [0.79, 1.32] | 0.85 | aOR = 1.20 [0.79, 1.81] | 0.39 |
|  | Other/Mixed/Unknown | OR = 1.09 [0.79, 1.48] | 0.58 | aOR = 1.24 [0.74, 2.03] | 0.41 |
| Insurance Type | Commercial | Ref | - | Ref | - |
|  | Medicare | OR = 0.59 [0.40, 0.84] | 0.005 | aOR = 0.90 [0.48, 1.65] | 0.74 |
|  | Medicaid/Public | OR = 0.88 [0.63, 1.21] | 0.44 | aOR = 0.83 [0.49, 1.37] | 0.47 |
|  | Self-Pay | OR = 1.18 [0.94, 1.48] | 0.16 | aOR = 1.02 [0.71, 1.47] | 0.92 |
| Any Mental Health Diagnosis | No | Ref | - | Ref | - |
|  | Yes | OR = 0.56 [0.46, 0.69] | <0.001 | aOR = 0.43 [0.31, 0.61] | <0.001 |
| Other Substance-Related Diagnosis | No | Ref | - | Ref | - |
|  | Yes | OR = 5.50 [4.05, 7.67] | <0.001 | aOR = 5.29 [3.46, 8.26] | <0.001 |
| MOUD Prescription 6 Months Prior | No MOUD | Ref | - | Ref | - |
|  | Any MOUD | OR = 122.58 [90.94, 167.07] | <0.001 | aOR = 132.23 [94.77, 187.57] | <0.001 |
| Overdose-Related Encounter | No overdose encounter | Ref | - | Ref | - |
|  | Overdose encounter | OR = 0.30 [0.16, 0.51] | <0.001 | aOR = 0.33 [0.15, 0.68] | 0.004 |
| Charlson Comorbidity Index | Per 1-point increase | OR = 0.91 [0.88, 0.93] | <0.001 | aOR = 0.94 [0.89, 0.99] | 0.01 |

# Supplementary Table S3. Sensitivity Analysis: Multivariable Logistic Regression Model Excluding Overdose-Related Encounter Among Patients With Opioid-Related Diagnoses and/or MOUD During Acute Care Encounter or Overdose-Related Encounter (N = 3,919)

|  |  | **Unadjusted Model** |  | **Adjusted Model** |  |
| --- | --- | --- | --- | --- | --- |
| **Variable** | **Level** | **Unadjusted Model (OR [95% CI])** | **p-value (Unadjusted)** | **Adjusted Model (aOR [95% CI])** | **p-value (Adjusted)** |
| Age Group | 18-34 | Ref | - | Ref | - |
|  | 35-49 | OR = 0.95 [0.73, 1.25] | 0.73 | aOR = 1.07 [0.69, 1.65] | 0.76 |
|  | 50-64 | OR = 0.78 [0.59, 1.03] | 0.08 | aOR = 1.07 [0.66, 1.74] | 0.77 |
|  | 65+ | OR = 0.57 [0.42, 0.77] | <0.001 | aOR = 1.10 [0.61, 1.97] | 0.75 |
| Sex | Male | Ref | - | Ref | - |
|  | Female | OR = 0.87 [0.70, 1.06] | 0.17 | aOR = 1.20 [0.86, 1.68] | 0.29 |
| Marital Status | Single | Ref | - | Ref | - |
|  | Married/Partnered | OR = 0.75 [0.58, 0.95] | 0.02 | aOR = 1.09 [0.71, 1.65] | 0.70 |
|  | Previously Married/Other | OR = 0.55 [0.40, 0.73] | <0.001 | aOR = 0.75 [0.46, 1.23] | 0.26 |
| Race/Ethnicity | Non-Hispanic White | Ref | - | Ref | - |
|  | Non-Hispanic Black | OR = 1.26 [0.91, 1.71] | 0.15 | aOR = 1.70 [1.02, 2.77] | 0.04 |
|  | Hispanic/Latinx | OR = 1.02 [0.79, 1.32] | 0.85 | aOR = 1.18 [0.78, 1.77] | 0.44 |
|  | Other/Mixed/Unknown | OR = 1.09 [0.79, 1.48] | 0.58 | aOR = 1.23 [0.73, 2.02] | 0.43 |
| Insurance Type | Commercial | Ref | - | Ref | - |
|  | Medicare | OR = 0.59 [0.40, 0.84] | 0.005 | aOR = 0.93 [0.50, 1.69] | 0.80 |
|  | Medicaid/Public | OR = 0.88 [0.63, 1.21] | 0.44 | aOR = 0.79 [0.47, 1.31] | 0.37 |
|  | Self-Pay | OR = 1.18 [0.94, 1.48] | 0.16 | aOR = 1.00 [0.69, 1.44] | 0.99 |
| Any Mental Health Diagnosis | No | Ref | - | Ref | - |
|  | Yes | OR = 0.56 [0.46, 0.69] | <0.001 | aOR = 0.44 [0.31, 0.61] | <0.001 |
| Other Substance-Related Diagnosis | No | Ref | - | Ref | - |
|  | Yes | OR = 5.50 [4.05, 7.67] | <0.001 | aOR = 5.44 [3.56, 8.49] | <0.001 |
| MOUD Prescription 6 Months Prior | No MOUD | Ref | - | Ref | - |
|  | Any MOUD | OR = 122.58 [90.94, 167.07] | <0.001 | aOR = 133.01 [95.55, 188.18] | <0.001 |
| Charlson Comorbidity Index | Per 1-point increase | OR = 0.91 [0.88, 0.93] | <0.001 | aOR = 0.94 [0.89, 0.99] | 0.02 |

# Supplementary Table S4. Sensitivity Analysis: Multivariable Logistic Regression Model Using Restrictive EHR-Based Opioid-Related Involvement Definition Among Patients With Opioid-Related Diagnoses and/or Overdose-Related Encounter Documented in the EHR (N = 3,585)

|  |  | **Unadjusted Model** |  | **Adjusted Model** |  |
| --- | --- | --- | --- | --- | --- |
| **Variable** | **Level** | **Unadjusted Model (OR [95% CI])** | **p-value (Unadjusted)** | **Adjusted Model (aOR [95% CI])** | **p-value (Adjusted)** |
| Age Group | 18-34 | Ref | - | Ref | - |
|  | 35-49 | OR = 0.88 [0.51, 1.53] | 0.65 | aOR = 0.68 [0.34, 1.39] | 0.29 |
|  | 50-64 | OR = 0.67 [0.37, 1.20] | 0.18 | aOR = 0.68 [0.31, 1.51] | 0.35 |
|  | 65+ | OR = 0.79 [0.44, 1.40] | 0.41 | aOR = 0.69 [0.27, 1.73] | 0.43 |
| Sex | Male | Ref | - | Ref | - |
|  | Female | OR = 0.87 [0.57, 1.32] | 0.51 | aOR = 0.86 [0.49, 1.48] | 0.58 |
| Marital Status | Single | Ref | - | Ref | - |
|  | Married/Partnered | OR = 0.85 [0.51, 1.38] | 0.52 | aOR = 0.78 [0.39, 1.50] | 0.46 |
|  | Previously Married/Other | OR = 0.66 [0.35, 1.15] | 0.16 | aOR = 0.87 [0.38, 1.94] | 0.75 |
| Race/Ethnicity | Non-Hispanic White | Ref | - | Ref | - |
|  | Non-Hispanic Black | OR = 1.04 [0.51, 1.94] | 0.90 | aOR = 1.26 [0.51, 2.91] | 0.60 |
|  | Hispanic/Latinx | OR = 0.86 [0.49, 1.43] | 0.57 | aOR = 1.10 [0.56, 2.12] | 0.78 |
|  | Other/Mixed/Unknown | OR = 0.99 [0.50, 1.81] | 0.98 | aOR = 1.09 [0.47, 2.41] | 0.83 |
| Insurance Type | Commercial | Ref | - | Ref | - |
|  | Medicare | OR = 1.12 [0.58, 2.06] | 0.71 | aOR = 1.47 [0.59, 3.55] | 0.40 |
|  | Medicaid/Public | OR = 1.12 [0.57, 2.08] | 0.73 | aOR = 1.03 [0.44, 2.31] | 0.94 |
|  | Self-Pay | OR = 1.25 [0.76, 2.03] | 0.37 | aOR = 0.98 [0.53, 1.80] | 0.95 |
| Any Mental Health Diagnosis | No | Ref | - | Ref | - |
|  | Yes | OR = 1.28 [0.83, 2.03] | 0.28 | aOR = 1.26 [0.71, 2.26] | 0.44 |
| Other Substance-Related Diagnosis | No | Ref | - | Ref | - |
|  | Yes | OR = 0.72 [0.48, 1.08] | 0.11 | aOR = 0.61 [0.35, 1.06] | 0.08 |
| MOUD Prescription 6 Months Prior | No MOUD | Ref | - | Ref | - |
|  | Any MOUD | OR = 132.49 [78.72, 233.40] | <0.001 | aOR = 138.30 [81.11, 247.04] | <0.001 |
| Overdose-Related Encounter | No overdose encounter | Ref | - | Ref | - |
|  | Overdose encounter | OR = 0.66 [0.26, 1.40] | 0.33 | aOR = 0.74 [0.25, 1.91] | 0.56 |
| Charlson Comorbidity Index | Per 1-point increase | OR = 0.98 [0.93, 1.03] | 0.50 | aOR = 0.96 [0.88, 1.03] | 0.23 |

# Supplementary Table S5. Sensitivity Analysis: Multivariable Logistic Regression Model Excluding Overdose-Related Encounter Using Restrictive EHR-Based Opioid-Related Involvement Definition Among Patients With Opioid-Related Diagnoses and/or Overdose-Related Encounter Documented in the EHR (N = 3,585)

|  |  | **Unadjusted Model** |  | **Adjusted Model** |  |
| --- | --- | --- | --- | --- | --- |
| **Variable** | **Level** | **Unadjusted Model (OR [95% CI])** | **p-value (Unadjusted)** | **Adjusted Model (aOR [95% CI])** | **p-value (Adjusted)** |
| Age Group | 18-34 | Ref | - | Ref | - |
|  | 35-49 | OR = 0.88 [0.51, 1.53] | 0.65 | aOR = 0.69 [0.34, 1.39] | 0.30 |
|  | 50-64 | OR = 0.67 [0.37, 1.20] | 0.18 | aOR = 0.69 [0.31, 1.53] | 0.36 |
|  | 65+ | OR = 0.79 [0.44, 1.40] | 0.41 | aOR = 0.70 [0.27, 1.75] | 0.44 |
| Sex | Male | Ref | - | Ref | - |
|  | Female | OR = 0.87 [0.57, 1.32] | 0.51 | aOR = 0.86 [0.49, 1.49] | 0.60 |
| Marital Status | Single | Ref | - | Ref | - |
|  | Married/Partnered | OR = 0.85 [0.51, 1.38] | 0.52 | aOR = 0.78 [0.39, 1.51] | 0.46 |
|  | Previously Married/Other | OR = 0.66 [0.35, 1.15] | 0.16 | aOR = 0.86 [0.37, 1.90] | 0.72 |
| Race/Ethnicity | Non-Hispanic White | Ref | - | Ref | - |
|  | Non-Hispanic Black | OR = 1.04 [0.51, 1.94] | 0.90 | aOR = 1.26 [0.52, 2.91] | 0.60 |
|  | Hispanic/Latinx | OR = 0.86 [0.49, 1.43] | 0.57 | aOR = 1.09 [0.55, 2.09] | 0.81 |
|  | Other/Mixed/Unknown | OR = 0.99 [0.50, 1.81] | 0.98 | aOR = 1.08 [0.47, 2.39] | 0.84 |
| Insurance Type | Commercial | Ref | - | Ref | - |
|  | Medicare | OR = 1.12 [0.58, 2.06] | 0.71 | aOR = 1.48 [0.60, 3.58] | 0.39 |
|  | Medicaid/Public | OR = 1.12 [0.57, 2.08] | 0.73 | aOR = 1.01 [0.44, 2.26] | 0.97 |
|  | Self-Pay | OR = 1.25 [0.76, 2.03] | 0.37 | aOR = 0.98 [0.53, 1.78] | 0.94 |
| Any Mental Health Diagnosis | No | Ref | - | Ref | - |
|  | Yes | OR = 1.28 [0.83, 2.03] | 0.28 | aOR = 1.25 [0.71, 2.25] | 0.45 |
| Other Substance-Related Diagnosis | No | Ref | - | Ref | - |
|  | Yes | OR = 0.72 [0.48, 1.08] | 0.11 | aOR = 0.62 [0.35, 1.06] | 0.08 |
| MOUD Prescription 6 Months Prior | No MOUD | Ref | - | Ref | - |
|  | Any MOUD | OR = 132.49 [78.72, 233.40] | <0.001 | aOR = 138.95 [81.51, 248.17] | <0.001 |
| Charlson Comorbidity Index | Per 1-point increase | OR = 0.98 [0.93, 1.03] | 0.50 | aOR = 0.96 [0.88, 1.03] | 0.23 |
